# Large-scale brain functional network abnormalities in social anxiety disorder

**DOI:** 10.1017/S0033291722003439

**Published:** 2023-10

**Authors:** Xun Zhang, Xun Yang, Baolin Wu, Nanfang Pan, Min He, Song Wang, Graham J. Kemp, Qiyong Gong

**Affiliations:** 1 Huaxi MR Research Center (HMRRC), Department of Radiology, West China Hospital of Sichuan University, Chengdu, Sichuan 610041, China; 2 Functional & Molecular Imaging Key Laboratory of Sichuan Province, West China Hospital of Sichuan University, Chengdu, Sichuan 610041, China; 3 Research Unit of Psychoradiology, Chinese Academy of Medical Sciences, Chengdu, Sichuan 610041, China; 4 School of Public Affairs, Chongqing University, Chongqing 400044, China; 5 Liverpool Magnetic Resonance Imaging Centre (LiMRIC) and Institute of Life Course and Medical Sciences, University of Liverpool, Liverpool L69 3BX, UK; 6 Department of Radiology, West China Xiamen Hospital of Sichuan University, Xiamen, Fujian 361000, China

**Keywords:** Functional network connectivity, independent component analysis, magnetic resonance imaging, psychoradiology, resting-state networks, social anxiety disorder, support vector machine

## Abstract

**Background:**

Although aberrant brain regional responses are reported in social anxiety disorder (SAD), little is known about resting-state functional connectivity at the macroscale network level. This study aims to identify functional network abnormalities using a multivariate data-driven method in a relatively large and homogenous sample of SAD patients, and assess their potential diagnostic value.

**Methods:**

Forty-six SAD patients and 52 demographically-matched healthy controls (HC) were recruited to undergo clinical evaluation and resting-state functional MRI scanning. We used group independent component analysis to characterize the functional architecture of brain resting-state networks (RSNs) and investigate between-group differences in intra-/inter-network functional network connectivity (FNC). Furtherly, we explored the associations of FNC abnormalities with clinical characteristics, and assessed their ability to discriminate SAD from HC using support vector machine analyses.

**Results:**

SAD patients showed widespread intra-network FNC abnormalities in the default mode network, the subcortical network and the perceptual system (i.e. sensorimotor, auditory and visual networks), and large-scale inter-network FNC abnormalities among those high-order and primary RSNs. Some aberrant FNC signatures were correlated to disease severity and duration, suggesting pathophysiological relevance. Furthermore, intrinsic FNC anomalies allowed individual classification of SAD *v.* HC with significant accuracy, indicating potential diagnostic efficacy.

**Conclusions:**

SAD patients show distinct patterns of functional synchronization abnormalities both within and across large-scale RSNs, reflecting or causing a network imbalance of bottom-up response and top-down regulation in cognitive, emotional and sensory domains. Therefore, this could offer insights into the neurofunctional substrates of SAD.

## Introduction

Social anxiety disorder (SAD) is characterized by disproportionate fear, anxiety and avoidance behaviour in social/performance situations (Stein & Stein, 2008), resulting in various emotional, cognitive and behavioural disabilities (Ruscio et al., 2008). Lifetime prevalence is 7–12% (Stein & Stein, 2008), often with comorbid psychopathology such as other anxiety disorders, major depressive disorder and substance abuse (Meier et al., 2015). SAD is typically chronic, and therapeutic options are limited (Penninx, Pine, Holmes, & Reif, 2021). This has prompted research into its neurobiological underpinnings, where the non-invasive methods of magnetic resonance imaging (MRI), especially functional MRI (fMRI), are particularly useful (Bas-Hoogendam et al., 2022; Zugman et al., 2022). This approach has demonstrated hyper-activation of the fronto-limbic circuitry (‘fear circuitry’) of prefrontal cortex (PFC), anterior cingulate cortex (ACC), insula and amygdala (Etkin & Wager, 2007), and more recently hyper-activation also of medial parietal and occipital regions (posterior cingulate, precuneus and cuneus) and hypoconnectivity of parietal, limbic and executive network regions (Bruhl, Delsignore, Komossa, & Weidt, 2014). This work supports a model in which dysfunctional bottom-up response and top-down regulation underlie the emotional hyper-arousal and impaired cognitive processing characteristic of SAD (Bas-Hoogendam & Westenberg, 2020; Bruhl et al., 2014; Etkin, 2012; Gentili et al., 2016).

Nevertheless, most of this evidence has come from task-fMRI, while it remains to be elucidated whether a similar pattern of functional alterations occurs in resting-state brain physiology as probed by resting-state fMRI (rs-fMRI), which offers a distinct perspective on the intrinsic neurobiology of SAD free from potential confounding effects of task performance (Smitha et al., 2017; Zhang et al., 2022a). In a recent systematic review of rs-fMRI studies of SAD (Mizzi, Pedersen, Lorenzetti, Heinrichs, & Labuschagne, 2022), the most consistent findings were aberrant activity in frontal regions and abnormal connectivity between frontal lobe and amygdala/parietal regions, suggesting that the classic model (Bruhl et al., 2014; Etkin & Wager, 2007) based on task-fMRI does not completely explain the resting-state neurobiology of SAD, and highlighting the need for more rs-fMRI studies with larger, homogenous samples and consistent analytic approaches (Mizzi et al., 2022).

Besides, it is increasingly recognized that the brain works as a system of interacting information-sharing networks (Damoiseaux et al., 2006; Lai et al., 2022). Functional connectivity (FC), indexing the temporal coherence of the haemodynamic activity of spatially remote brain areas (Biswal, Yetkin, Haughton, & Hyde, 1995), is widely used to characterize functional interactions among brain networks (Suo et al., 2022; Van Dijk et al., 2010), with two main technical approaches: seed/region of interest (ROI)-based calculation and independent component analysis (ICA). The first approach is vulnerable to the variability of ROI location, size and shape, as well as inter-subject anatomical variation (He et al., 2016), to which the multivariate data-driven method of ICA, which can identify a battery of maximally spatially-independent but temporally-coherent components (i.e. intrinsic functional networks), is largely immune (Calhoun, Adali, Pearlson, & Pekar, 2001). Hence, characterizing intra- and inter-network functional network connectivity (FNC) using ICA is a powerful tool to probe macroscale functional integration/dissociation in the normal and abnormal brain (Cai et al., 2021; Fox & Raichle, 2007; Houck et al., 2017; Huang et al., 2018). However, to the best of our knowledge, only three studies have used ICA and rs-fMRI in SAD; one investigated the intrinsic FNC as candidate endophenotype in families genetically enriched for SAD, in which most (22/39) subjects were diagnosed with subclinical SAD, and had psychiatric comorbidity (Bas-Hoogendam, van Steenbergen, Cohen Kadosh, Westenberg, & van der Wee, 2021); the other two studies had relatively small sample size (18 and 20 SAD patients) (Geiger et al., 2016; Liao et al., 2010).

In this study, we acquired rs-fMRI data and used ICA to characterize intrinsic intra-/inter-network FNC abnormalities in a relatively large and homogenous sample of adult subjects with SAD (*n* = 46). We also explored the associations of FNC abnormalities with clinical characteristics. Lastly, we used machine learning to investigate the potential diagnostic efficacy of those FNC signatures. We hypothesized that: (i) SAD patients, compared with healthy controls (HC), would show FNC impairments mainly in default mode network (DMN), frontal parietal network (FPN), salience network (SN) and subcortical network (SCN), previously shown to be abnormal in SAD (Bas-Hoogendam et al., 2021; Geiger et al., 2016; Liao et al., 2010; Mizzi et al., 2022; Xu et al., 2019; Yang et al., 2019); (ii) altered FNC would be related to clinical features (e.g. symptom severity); and (iii) intrinsic FNC markers would have good sensitivity and specificity for SAD diagnosis.

This study is a follow-up analysis of data from a previously-reported cohort (Zhang et al., 2022b), with different aims and methods. The present study used data-driven methods (ICA) to characterize whole-brain resting-state networks (RSNs) and explore intra-/inter-network FNC abnormalities at the level of large-scale networks; by contrast the previous study aimed to determine whole-brain voxel-wise FC abnormalities based on predefined ROIs which were brain regions with grey matter volume deficits, driven by the hypothesis that brain structural abnormalities may give rise to clinical syndromes via disruption of FC (Gong, 2020; Lui, Zhou, Sweeney, & Gong, 2016).

## Methods

### Participants

We recruited 49 right-handed adult SAD patients without any comorbid psychiatric disorders from the Mental Health Center of the West China Hospital at Sichuan University. In accordance with the criteria of Diagnostic and Statistical Manual of Mental Disorders, Fourth Edition (DSM-IV), the diagnosis of SAD was established by two experienced clinical psychiatrists through the Structured Clinical Interview for DSM Disorders (SCID). According to the power analysis using G Power software (Faul, Erdfelder, Lang, & Buchner, 2007), a medium-sized effect with adequate statistical power (Cohen's *d* = 0.5, *α* = 0.05, 1–*β* = 0.8) using an independent-sample *t* test required at least 102 subjects. Considering this, we recruited 53 demographically-matched (i.e. sex, age and handedness) HC from the local community for comparison analysis, using the SCID-Non-Patient Version to confirm the lifetime absence of psychiatric and neurological diseases. The exclusion criteria for all participants were: (1) comorbidity with other axis I psychiatric disorders, axis II antisocial or borderline personality disorders (verified by SCID); (2) currently receiving psychopharmacological/psychological therapy; (3) history of substance dependence or abuse; (4) learning or developmental disorders; (5) history of head injury; (6) current major neurological or physical diseases; (7) family history of mental disorders; and (8) current pregnancy, claustrophobia or other contraindications to MRI examination. Individuals were also excluded if they were aged under 18 or over 60 years, to minimize age-related effects. Notably, several other analyses of MRI data from these participants have been reported: resting-state functional network-based statistic and graph-theory analyses (Yang et al., 2019), analyses of cortical thickness and surface area (Zhang et al., 2020) and grey matter volume and seed-based FC analyses (Zhang et al., 2022b), with the results reported in the cited papers.

Illness duration was defined as the period between the first reported/observed alterations in psychological/behaviour state to the development of disease when the patients participated in the study (Singh et al., 2005), information being provided by patients, family members and medical records. Social anxiety was evaluated with the self-reported Liebowitz Social Anxiety Scale (LSAS) (Mennin et al., 2002), the most commonly-used clinical scale in SAD studies; the 24-item LSAS provides scores for fear factor (LSASF) and social avoidance factor (LSASA), their sum being the total score (LSAST). LSAS has shown good validity and reliability in Chinese populations (He & Zhang, 2004).

All procedures complied with the ethical standards of the relevant national and institutional committees on human experimentation and with the Helsinki Declaration of 1975, as revised in 2008. This study was approved by the Medical Research Ethics Committee of West China Hospital at Sichuan University. After a full explanation of all procedures, all subjects provided written informed consent to participate.

### Image acquisition and pre-processing

#### Image acquisition

This study used an SAD dataset in which we acquired high-resolution three-dimensional T1-weighted images, rs-fMRI data and diffusion tensor imaging sequentially on a 3.0 T MR scanner (Siemens Trio, Erlangen, Germany) with a 12-channel head coil. Before the scans, the subjects were instructed to lie still, keep their eyes closed and to stay relaxed but awake. Earplugs were used to reduce scanner noise, and foam pads to minimize head motion. High-resolution three-dimensional T1-weighted images were acquired with a spoiled gradient-recalled sequence with the following parameters: repetition time (TR)/echo time (TE) 1900 ms/2.26 ms, flip angle 9°, 176 sagittal slices, slice thickness 1 mm, field of view (FOV) 240 × 240 mm^2^, data matrix 256 × 256, voxel size 1 × 1 × 1 mm^3^, in-plane resolution 0.94 × 0.94 mm^2^. The rs-fMRI data were obtained with a gradient echo-planar imaging sequence: TR/TE 2000 ms/30 ms; flip angle 90°; acquisition matrix 64 × 64; FOV 240 × 240 mm^2^; thickness 5.0 mm, without gap; voxel size 3.75 × 3.75 × 5 mm^3^; 205 volumes. Each scan was inspected by an experienced neuroradiologist to rule out visible artefacts and lesions.

#### Image pre-processing

First, the rs-fMRI data were pre-processed using the Data Processing Assistant for Resting-State fMRI (DPARSF 4.3, http://rfmri.org/DPARSF), which is based on Statistical Parametric Mapping software (SPM12; Welcome Department of Cognitive Neurology, London, UK; http://www.fil.ion.ucl.ac.uk/spm/) (Ashburner & Friston, 2005) and the toolbox for Data Processing and Analysis of Brain Imaging (DPABI, http://rfmri.org/DPABI) (Yan, Wang, Zuo, & Zang, 2016). Briefly, this includes (1) removal of the first 10 volumes and slice timing correction; (2) realignment and correction for head motion (three SAD patients and one HC with head motion above 2.5 mm or 2.5° in any direction were excluded), in which we also calculated the frame-wise displacement (FD) to summarize the head motion; (3) spatial normalization to Montreal Neurological Institute space including the new segmentation and Diffeomorphic Anatomical Registration Through Exponentiated Lie algebra (DARTEL) (Ashburner, 2007); (4) resampling into 3 × 3 × 3 mm^3^ and spatial smoothing with a 8 mm full-width at half-maximum Gaussian kernel.

#### Independent component analysis

**Fig. 1.**
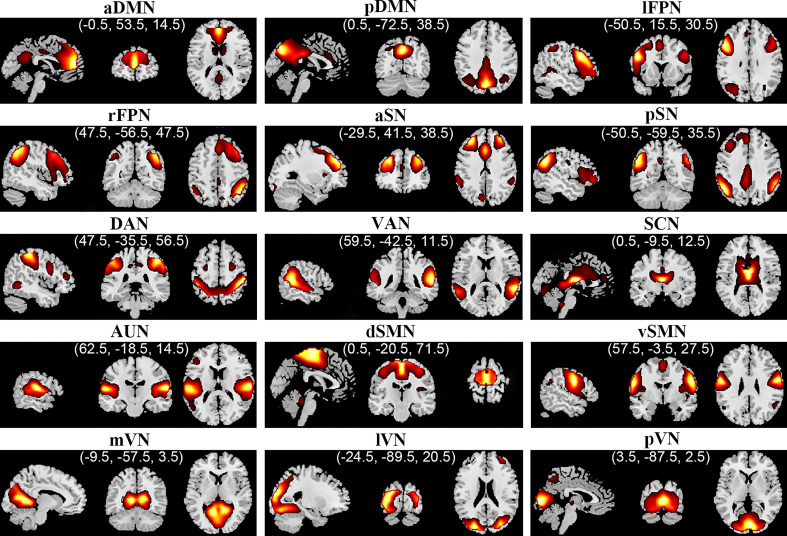
Spatial maps of 15 selected independent components. In parentheses are the peak coordinates (X, Y, Z) of corresponding components. Abbreviations: aDMN, anterior default mode network; aSN, anterior salience network; AUN, auditory network; DAN, dorsal attention network; dSMN, dorsal sensorimotor network; lFPN, left frontoparietal network; lVN, lateral visual network; mVN, medial visual network; pDMN, posterior default mode network; pSN, posterior salience network; pVN, posterior visual network; rFPN, right frontoparietal network; SCN, subcortical network; VAN, ventral attention network; vSMN, ventral sensorimotor network.

Spatial group ICA (GICA) was performed to parcellate the rs-fMRI data using GIFT software (http://mialab.mrn.org/software/gift/), and the number of independent components (ICs) was estimated automatically by the software, in which spatial GICA decompose the individual data into spatial ICs with a unique time course profile (Kiviniemi et al., 2009). Briefly: (1) Individual rs-fMRI data were decomposed through principal component analysis for dimension reduction into principal components. (2) The infomax algorithm was applied to the reduced data of all participants to concatenate across time and decompose data, the concatenated subject-reduced data being decomposed into 24 ICs: this algorithm was repeated 20 times using ICASSO (http://research.ics.tkk.fi/ica/icasso/) to improve estimation reliability, selecting the most central run for further analyses (Himberg, Hyvärinen, & Esposito, 2004). (3) A GICA back-reconstruction approach was used to produce subject-specific time courses and spatial ICs maps (Allen, Erhardt, Wei, Eichele, & Calhoun, 2012). The ICs were identified as meaningful if they had peak activations in grey matter with low spatial overlap with known vascular, ventricular, motion, susceptibility artefacts and edges, in addition to the domination of low-frequency power (Allen et al., 2014). To sort the meaningful ICs into different RSNs, RSNs templates were used as the reference for multiple regression with the ICs using the ‘sort components’ tool of the GIFT toolbox, in which greater regression coefficient indicates more similarity to the RSNs (Liao et al., 2010; Wang et al., 2020a). Eventually, 15 out of 24 ICs were characterized as 15 RSNs (Fig. 1), including anterior and posterior default mode network (aDMN and pDMN); left and right frontoparietal network (lFPN and rFPN); dorsal and ventral attention network (DAN and VAN); anterior and posterior salience network (aSN and pSN); SCN; auditory network (AUN); dorsal and ventral sensorimotor network (dSMN and vSMN); medial, lateral and posterior visual network (mVN, lVN and pVN). (4) Finally, before network-wise FC analyses, time courses of identified RSNs underwent additional post-processing procedures including de-trending linear, quadratic and cubic trends; de-spiking detected outliers; and low-pass filtering with a cut-off of 0.15 Hz (Wang et al., 2020a).

### Network-wise functional connectivity analyses

#### Intra-network FNC analyses

Intra-network connectivity, representing the contribution of the time course to each voxel comprising a corresponding component (Wang et al., 2021), was calculated using the spatial maps. Specifically, all subjects' spatial maps for each RSN were entered into a random-effect one-sample *t* test. Brain regions were identified within each corresponding RSN when they met a threshold of family-wise error (FWE)-corrected *p* < 0.001 in combination with a proper extent threshold for multiple comparisons. Then, voxel-wise comparisons of intra-network FNC between SAD patients and HC were performed using independent-sample *t* test with age, sex and mean FD as covariates of no interest in SPM12. The FWE approach was used to control for multiple comparisons with a significance threshold of voxel-wise *p* < 0.001 and FWE-corrected *p* < 0.05 at cluster level (Hayasaka & Nichols, 2003; Woo, Krishnan, & Wager, 2014).

#### Inter-network FNC analyses

Inter-network FNC was computed as the correlation coefficient between the time courses of the RSNs, thus constructing a symmetric 15 × 15 inter-network FNC matrix for each individual. The FNC matrices were *z*-normalized using Fisher's *r*-to-*z* transformation to improve the normality of the partial correlation coefficients. Finally, between-group differences of inter-network FNC were compared using independent-sample *t* test after controlling for age, sex and mean FD. The false discovery rate (FDR) approach was used to correct for multiple comparisons with a significance threshold of *p* < 0.05 (Benjamini & Yekutieli, 2001).

#### Clinical relevance analyses

To identify the relationships between the intrinsic FNC impairments and clinical features, the intra-/inter-network FNC values with significant between-group differences were extracted respectively, then partial correlation analyses were conducted between the aforementioned FNC values and clinical characteristics (i.e. LSAST, LSASA, LSASF and disease duration) with sex, age and mean FD as covariates in the SAD group, using IBM SPSS Statistics 22.0.

#### Machine learning analyses

To explore the potential diagnostic value of intrinsic FNC with significant between-group differences, support vector machine (SVM) analyses (Cortes & Vapnik, 1995) were conducted to investigate how well FNC could differentiate SAD *v.* HC at the individual level. In brief: (1) Each subject's average intra-network FNC of each cluster and inter-network FNC values that showed significant between-group differences were regarded as features for model training. (2) Leave-one-out cross-validation was used to separate training and testing sets. (3) Data normalization was performed on the feature matrix to guarantee that the features were at the same magnitude for subsequent analyses. (4) The SVM optimal hyperparameter (i.e. soft margin parameter C) was selected on the training sets. (5) The SVM classification algorithm with a linear kernel was used to determine the hyperplane maximizing the margin between binary classes in the feature space, and the classification strategy learned from the training sample was used to predict individual classification in testing sets. (6) The classification performance of the model was assessed using sensitivity, specificity and total accuracy based on testing sets. The receiver operating characteristic (ROC) curve was also constructed, in which the area under the ROC curve (AUC) was calculated for quantification. (7) Non-parametric permutation test (5000 times) was applied to estimate statistical significance for the machine learning model. All these procedures were conducted in LIBSVM for support vector classification (Chang & Lin, 2011). More details are available in online Supplementary Materials.

## Results

### Demographic and clinical characteristics

There were no significant group differences (SAD patients *v.* HC) in sex composition and age; as expected, SAD patients scored significantly higher on LSAS (Table 1). There were no significant group differences in mean FD [*t* = 0.519, *p* = 0.605].

**Table 1.** Demographics and clinical characteristics of participants

Characteristics	SAD (*N* = 46)	HC (*N* = 52)	*p* value
Sex (Male/Female)	28/18	30/22	0.749^a^
Age (years)	24.8 ± 5.3	23.3 ± 3.1	0.07^b^
Illness duration (years)	7.1 ± 4.2	–	–
LSAST	65.6 ± 23.4	18.6 ± 8.5	<0.001^b^
LSASF	32.6 ± 11.6	10.3 ± 5.3	<0.001^b^
LSASA	32.6 ± 12.8	8.3 ± 6.1	<0.001^b^

HC, healthy controls; LSAST, LSASF and LSASA, total score and fear and avoidance factor scores on the Liebowitz Social Anxiety Scale (LSAS); SAD, social anxiety disorder.

Continuous variables are presented as the means ± standard deviations.

a *p* value obtained using a χ^2^ test.

b *p* value obtained using an independent-sample *t* test.

### Group differences in intra-network FNC

**Fig. 2.**
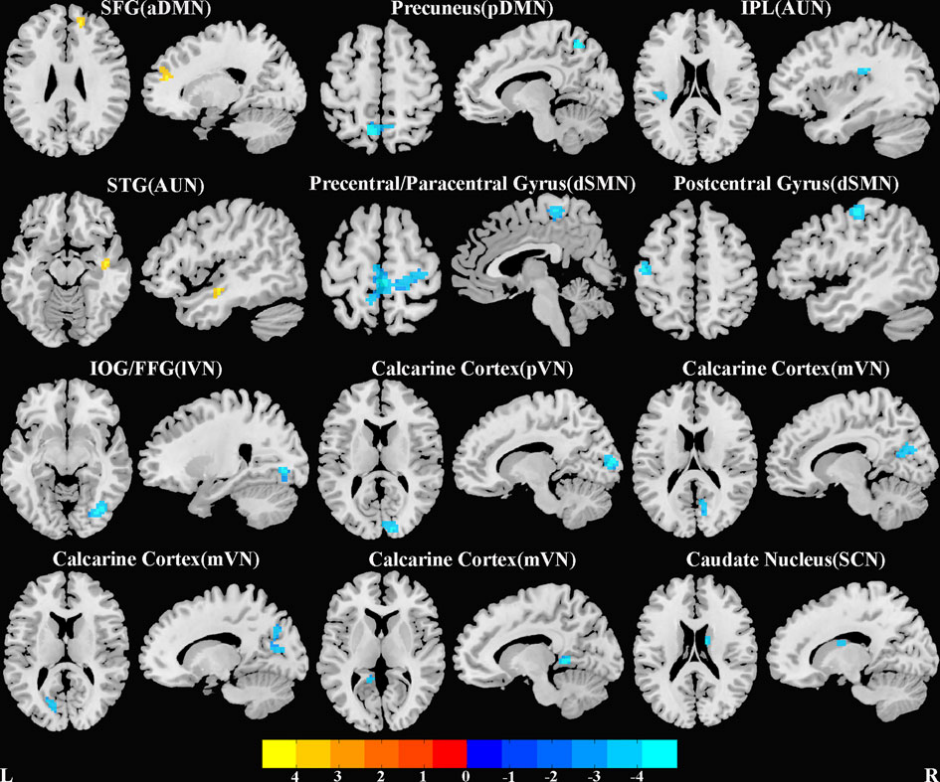
Brain regions with significant differences of intrinsic intra-network functional connectivity between SAD patients and HC. All clusters survived correction for multiple comparisons with a significance threshold of a voxel-wise value of *p* < 0.001 and a family-wise error-corrected *p* < 0.05 at cluster level. Warm colours (positive values) represent increased intrinsic functional connectivity, cooler colours (negative values) decreased intrinsic functional connectivity, in SAD patients compared to HC. Abbreviations: aDMN, anterior default mode network; AUN, auditory network; dSMN, dorsal sensorimotor network; FFG, fusiform gyrus; HC, healthy controls; IOG, inferior occipital gyrus; IPL, inferior parietal lobe; lVN, lateral visual network; mVN, medial visual network; pDMN, posterior default mode network; pVN, posterior visual network; SAD, social anxiety disorder; SCN, subcortical network; SFG, superior frontal gyrus; STG, superior temporal gyrus.

SAD patients, compared to HC, had significantly increased intra-network FNC in aDMN (right superior frontal gyrus, mainly in mPFC) and AUN [right superior temporal gyrus (STG)]. SAD patients had significantly decreased intra-network FNC in pDMN (left precuneus), AUN [left inferior parietal lobe (IPL)], dSMN (bilateral paracentral/right precentral gyrus and left postcentral gyrus), lVN [right inferior occipital gyrus (IOG)/fusiform gyrus (FFG)], mVN and pVN (mainly including bilateral calcarine cortex) and SCN (right caudate) (Fig. 2 and online Supplementary Table S1).

### Group differences in inter-network FNC

**Fig. 3.**
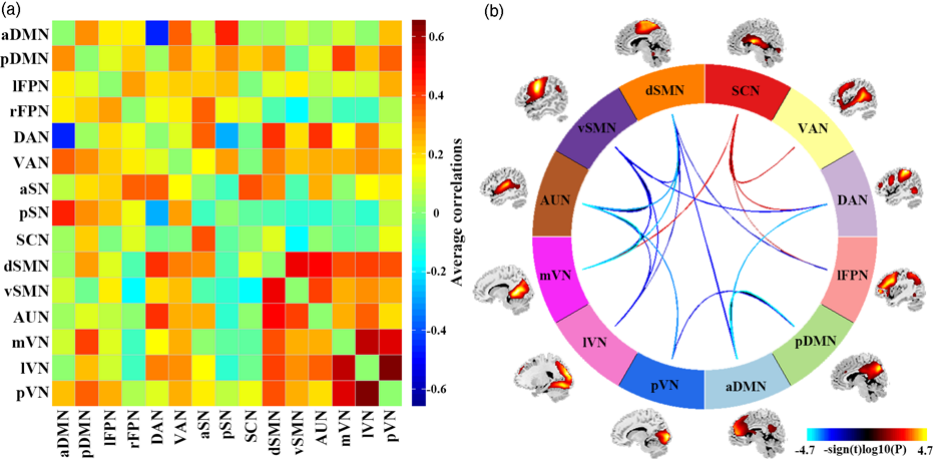
Results of inter-network functional connectivity analyses. (a) Inter-network functional connectivity matrix. Pairwise correlations between resting-state functional networks were averaged across participants. (b) Between-group differences of inter-network functional connectivity between SAD patients and HC. Warmer colours represent increased inter-network FNC, cooler colours decreased inter-network FNC in social anxiety disorder compared to healthy controls. Abbreviations: aDMN, anterior default mode network; aSN, anterior salience network; AUN, auditory network; DAN, dorsal attention network; dSMN, dorsal sensorimotor network; lFPN, left frontoparietal network; lVN, lateral visual network; mVN, medial visual network; pDMN, posterior default mode network; pSN, posterior salience network; pVN, posterior visual network; rFPN, right frontoparietal network; SCN, subcortical network; VAN, ventral attention network; vSMN, ventral sensorimotor network.

SAD patients, compared to HC, had significantly increased inter-network FNC between SCN and lFPN, DAN, VAN, mVN. SAD patients had significantly decreased inter-network FNC between aDMN and pDMN, DAN, dSMN; between pDMN and pVN; between lFPN and dSMN; between DAN and vSMN; between dSMN and vSMN, AUN, mVN, lVN; between vSMN and AUN, mVN, lVN; and between AUN and mVN, lVN, pVN (Fig. 3).

### Clinical correlates of intrinsic FNC

Among the 12 significant intra-network FNCs and 20 significant inter-network FNCs, after controlling for the confounders of sex, age and mean FD, the intra-network FNC of mVN (i.e. left calcarine cortex) was significantly positively correlated with illness duration (*r* = 0.313, *p* = 0.041); the inter-network FNC of vSMN-mVN was positively correlated with illness duration (*r* = 0.325, *p* = 0.034); significant positive correlations were also observed between inter-network FNC of mVN-SCN and LSAST (*r* = 0.317, *p* = 0.038) and also LSASA (*r* = 0.411, *p* = 0.006) (online Supplementary Fig. S1). None of these results survived correction for multiple tests at FDR-corrected *p* < 0.05.

### Single-subject classification of SAD patients *v.* HC

**Fig. 4.**
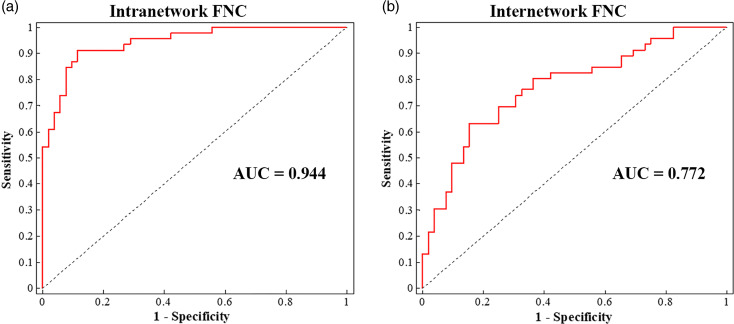
Single-subject classification of SAD patients *v.* HC. Abbreviations: AUC, area under the receiver operating characteristic curve; FNC, functional network connectivity; HC, healthy controls; SAD, social anxiety disorder.

The accuracy of SVM classification for SAD *v.* HC based on the significant intrinsic FNC was highly significantly above chance (*p* < 0.001); the intra-network FNC demonstrated the best performance with total accuracy 86.7%, sensitivity 91.3%, specificity 88.5% and AUC 94.4% (Fig. 4 and online Supplementary Table S2).

## Discussion

To the best of our knowledge, this is the first study to investigate intrinsic FNC at resting-state in a relatively large and homogeneous sample of patients with SAD. SAD patients had intra-network FNC abnormalities mainly in aDMN, pDMN, SCN and the perceptual system (SMN, AUN and VN) and large-scale inter-network FNC abnormalities among DMN, FPN, AN, SCN, SMN, VN and AUN. Abnormal FNC were correlated to SAD severity and duration, suggesting pathophysiological relevance. Furthermore, intrinsic FNC anomalies allowed individual classification of SAD *v.* HC with significant accuracy, suggesting potential diagnostic efficacy. These findings offer some insights into the neurobiology of SAD, as we discuss briefly below.

### Intra-network FNC abnormalities in SAD

At the intra-network level, we found increased intrinsic FNC in aDMN (mPFC), in combination with decreased FNC in pDMN (precuneus), which is broadly consistent with previous reports in SAD (Bas-Hoogendam, van Steenbergen, Tissier, van der Wee, & Westenberg, 2020; Heitmann et al., 2016; Liu et al., 2015b; Mizzi et al., 2022). The DMN plays a critical role in self/other-referential judgements, emotional processing, recollection of experiences and analysis of others' mental states (Raichle, 2015), and is involved in other social, affective and introspective processes (Amft et al., 2015): aDMN is mainly involved in self/other-referential judgements, while pDMN is implicated in autobiographical/episodic memory retrieval and scene construction (Xu, Yuan, & Lei, 2016). It is tempting to relate abnormal FNC in DMN to functional cognitive models of disturbed self-evaluative and referential processes, such as maladaptive self-focused attention, emotional hyperarousal in combination with defective top-down regulation, post-event rumination, excessive focus and unreasonable speculation on others' intentions and facial expressions (Cremers & Roelofs, 2016).

Besides, consistent with extant literature (Finlayson-Short, Harrison, & Davey, 2021; Hjorth et al., 2021; Xu et al., 2019), dysfunctional intrinsic connectivity in the caudate nucleus (ventral striatum) was observed in SAD patients. The striatum is linked to many important functions including motor and cognitive control, emotional regulation, social learning and reward-related motivation processing (Lago, Davis, Grillon, & Ernst, 2017; Pan et al., 2021; Pennartz et al., 2009); recruitment of reward system (including ventral striatum) is also involved in self-referential processing, particularly when external stimuli are considered to be self-related (Northoff & Hayes, 2011). In this sense, intrinsic FNC abnormality in caudate nucleus may underlie imbalance of the neural approach-avoidance motivation system and self-referential mental activity in SAD (Xu et al., 2019).

Further, we found abnormal intrinsic FNC in the perceptual/sensory system, mainly dSMN (bilateral paracentral/right precentral gyrus and left postcentral gyrus), AUN (right STG and left IPL) and VN [lVN (right IOG/FFG), mVN and pVN (bilateral calcarine cortex)]. Abnormal activity and connectivity involving SMN, AUN and VN is reported in SAD, both in response to socially threatening stimuli and at rest (Dixon et al., 2020; Goldin, Manber, Hakimi, Canli, & Gross, 2009; Liao et al., 2010; Liu et al., 2015a; Phan, Fitzgerald, Nathan, & Tancer, 2006; Zhang et al., 2022b), and in one report the response to pharmacotherapy was associated with altered neural activation of this perceptual/sensory system in a social anxiety imagery task (Kilts et al., 2006). The SMN is important in the analysis of others' communicative intentions from perceptual cues including gaze direction, body gesture and facial expression (Conty, Dezecache, Hugueville, & Grezes, 2012). Consequently, dysconnectivity in SMN may be related to gaze avoidance towards emotional stimuli in SAD (Weeks, Howell, & Goldin, 2013). Additionally, AUN is considered to be involved not only in auditory information perception and processing but also social cognition (mnemonic and attentional) of fearful experiences (Quirk, Armony, & LeDoux, 1997): for instance, STG (the core component of AUN) demonstrated aberrant activation and dysconnectivity in anxiety patients actively listening to threat-related words (Zhao, Xi, Wang, Li, & He, 2014). In this sense, our results of impaired intra-network connectivity in AUN may indicate abnormal auditory information perception and social cognitive processing in SAD. Furthermore, VN is crucial in social information processing: for example, the FFG, part of the lVN responsible for the perception of emotions in facial stimuli (Gomez et al., 2017) and facial emotion processing, is usually abnormal in SAD (Machado-de-Sousa et al., 2010). Our results therefore offer further evidence for perceptual impairments and compromised social information processing in SAD.

### Inter-network FNC abnormalities in SAD

An optimal balance between functional specialization (intra-network synchronization) and integration (inter-network coupling) during the dynamic interactions of multiple networks is essential to high-level affective and cognitive processes (Berman et al., 2016). Even for networks (e.g. FPN, AN) whose intrinsic intra-network connectivity did not alter in SAD, we found striking large-scale inter-network connectivity abnormalities.

The DMN consists of two subsystems (aDMN and pDMN) that interact with a common core system, of which the mPFC and PCC are the respective hubs (Andrews-Hanna, Reidler, Sepulcre, Poulin, & Buckner, 2010). The former is mainly involved in self/other-referential judgements, while the latter is implicated in autobiographical/episodic memory retrieval and scene construction (Xu et al., 2016). Proper coupling of aDMN and pDMN is vital for normal brain functioning (D'Argembeau et al., 2010), and this is aberrant in several neuropsychiatric disorders (Hare et al., 2019; Wang et al., 2020b; Zhang et al., 2015). Our finding of decreased FNC between aDMN and pDMN may reflect a disrupted functional coupling, underpinning dysfunctional DMN-related clinical manifestations in SAD (e.g. maladaptive self-evaluative events and referential processes).

The task-negative DMN is deactivated during goal-directed behaviour with focused attention (Raichle, 2015), whereas the anti-correlated task-positive DAN has a top-down role in managing rules and goals during externally directed tasks (Turner & Spreng, 2012). These systems work competitively, switching between internally and externally oriented cognitive processing (Fox et al., 2005), contributing to cognitive control, emotional regulation and episodic memory performance (Anticevic et al., 2012; Kragel & Polyn, 2015). Our finding of decreased FNC between aDMN and DAN may reflect disrupted switching between internally and externally oriented cognitive control and emotional regulation in SAD.

SAD patients demonstrated increased inter-network FNC between SCN and lFPN, DAN, VAN and mVN. In the latest neurocircuitry model, core characteristics of SAD are bottom-up hyper-response and top-down reduced regulation efficiency, leading to emotional hyper-arousal and diminished cognitive control (Bruhl et al., 2014). In support of this, hypo-activation in the high-order cortical areas and hyper-activation in the subcortical regions, as well as decreased FC of cortical–subcortical circuitry have been widely reported in SAD (Mizzi et al., 2022; Zhang et al., 2022b). Nevertheless, we found increased connectivity in the cortical–subcortical circuit (i.e. SCN-lFPN/DAN/VAN). As increased connectivity is usually taken to indicate increased coupling and integrated communication (i.e. enhanced potential for top-down control and modulation), an appealing explanation is that top-down control modulation is increased but still fails to compensate for heightened social anxiety, perhaps due to disturbed structural connectivity or insufficient strong enough control (Bruhl et al., 2014; Yang et al., 2019).

We also found dysconnectivity among higher-order cognitive control systems (e.g. DMN, lFPN, DAN) and the primary perceptual/sensorimotor system (e.g. mVN, lVN, pVN, dSMN, vSMN, AUN). SAD patients suffer from persistent cognitive biases regarding socially threatening cues, notably faces and voices (Morrison & Heimberg, 2013). There is substantial evidence for involvement of perceptual/sensorimotor system in emotion perception and experience (Hardee et al., 2017), perception of fear expression in faces (i.e. for processing social face signals) (Pourtois et al., 2004) and emotional regulation in responses to threat-related information (Kropf, Syan, Minuzzi, & Frey, 2019). Impaired processing for sensory/perceptual integration of audio-visual signals in corresponding sensorimotor cortices and disrupted cognitive modulation in the higher integrative networks is responsible for clinical signs of hypervigilance towards social stimuli, exaggerated fear responses and consequent avoidance behaviour (Kreifelts et al., 2019; Kreifelts et al., 2020). In short, abnormalities within and across sensorimotor/perceptual-cognitive interactions result in inappropriate processing of external social signal, abnormal emotional arousal and cognitive bias. It may be, therefore, that the decreased FNC between the cognitive-control and sensorimotor/perceptual systems underlies hypervigilance towards threateningly social stimuli, persistent heightened attentiveness to sensory input, disrupted perceptual analysis of sensory events and dysfunctional cognitive control in SAD (Miskovic & Schmidt, 2012).

These results make an interesting comparison with our previous seed-based rs-fMRI analysis in these patients (Zhang et al., 2022b). That demonstrated decreased FC between SCN with the components of DMN (PFC/ACC and cerebellum), with part of the SMN (supplementary motor area), and increased FC between SCN and temporal lobe (Zhang et al., 2022b); the present ICA-based study shows widespread inter-network FNC abnormalities among higher-order cognitive control systems (e.g. DMN, lFPN, DAN, VAN and SCN) and the primary perceptual/sensorimotor system (e.g. mVN, lVN, pVN, dSMN, vSMN, AUN). The likeliest reason for these seeming discrepancies is that the two analyses were conducted by different methods and at different levels of brain patterns: the seed-based approach investigates the single interaction between the predefined ROI and each whole-brain voxel, while the ICA investigates multiple simultaneous voxel-to-voxel interactions among large-scale networks (Smith et al., 2013; Smitha et al., 2017).

### FNC as a potential diagnostic biomarker for SAD

We present the first evidence, to our knowledge, that intrinsic abnormal FNC (especially the intra-network FNC) allows individual classification of SAD *v.* HC with significant accuracy. Machine learning is a promising tool to help clinicians develop neuroimaging-based biomarkers for early diagnosis in clinical practice (Chen et al., 2020; Frick et al., 2014; Liu et al., 2015a; Zhan et al., 2021), and could potentially guide early diagnosis and interventions to improve the quality of life of SAD patients (Bas-Hoogendam & Westenberg, 2020; Etkin, 2019). Considering the prevalent comorbidity of other neuropsychiatric disorders in SAD, future research should investigate whether our findings are selective for SAD, rather than trans-diagnostic characteristics of psychiatric disorders.

### Limitations and future directions

This study has several limitations. First, the cross-sectional design precludes it from explicit causal inference. This will need longitudinal studies recruiting both SAD patients and individuals with high susceptibility to developing SAD (e.g. based on the genotypes and endophenotypes (Bas-Hoogendam et al., 2016)). Second, it would have been desirable to measure (and use to match with HC) general cognitive ability (e.g. general intelligence). Nevertheless, as there is no definite evidence that SAD patients suffer from intellectual impairment (Stein & Stein, 2008), we do not expect the general intelligence is a significant confounder. Third, although medium-sized effects were expected based on power analysis (as far as we know, the present study is the relatively large single-centre study investigating intrinsic FNC deficits using ICA in non-comorbid SAD patients), our sample size is smaller than some recent studies in other psychiatric disorders. A major reason for this is the strictness of our inclusion criteria: we studied only adult SAD patients without any comorbid disorders, in the hope of probing the specific neurofunctional underpinnings of SAD, which may also restrict the generalizability of our findings. This will require specific investigation on the potential effects of those demographic confounded factors on intrinsic functional anomaly in SAD in the future, and our results need further replication via a larger sample. Fourth, considering that SAD typically evolves during late childhood and early adolescence, it remains unknown how applicable these findings on adults are to adolescents; establishing this will require similar studies of child and adolescent SAD patients. Fifth, our rs-fMRI findings showed considerable overlap with those of previous task-fMRI studies, indicating that some aberrant intrinsic network connectivity as disturbed function for SAD can also be observed at rest, yet it remains elusive how the two aspects relate, and this merits future study to investigate the exact relationship between intrinsic network connectivity from task-fMRI and rs-fMRI. Sixth, our machine learning results are more of a preliminary exploration on the potential diagnostic value of intrinsic FNC, and will need extending with external validation samples in future classification studies. Finally, it needs to be clarified whether current results could have been confounded by examination-related anxiety during the MRI scans; future studies could usefully evaluate the psychophysiological reactions of participants before, during and after the MRI examination.

## Conclusions

Using data-driven ICA and SVM analyses, this study identified in SAD patients intrinsic FNC abnormalities within and across large-scale brain functional networks involving not only the high-order cognitive networks (e.g. DMN, FPN, DAN, VAN and SCN) but also the primary perceptual networks (e.g. VN, SMN and AUN), some of which were correlated to symptom severity and disease duration. Aberrant intrinsic FNC might be useful to discriminate SAD from HC at individual level. This study offers some insights into the neurobiology of SAD, which may help to identify neurofunctional biomarkers for its clinical diagnosis.

## Data Availability

The data and code that support the findings of present study are available from the corresponding author through reasonable request. The data and code sharing adopted by the authors comply with the requirements of the funding institute and with institutional ethics approval.

## Supplementary material

For supplementary material accompanying this paper visit https://doi.org/10.1017/S0033291722003439. click here to view supplementary material
