# Supplementary material for: Large-scale brain functional network abnormalities in social anxiety disorder
Source: Psychol Med. 2022 Nov 4;53(13):6194–204. doi: 10.1017/S0033291722003439 (PMC10520603; doi:10.1017/S0033291722003439)
Supplement: Supplementary file 1 [file S0033291722003439sup001.docx]

# Online Supplementary Materials

### Methods

**Support vector machine analyses**

To investigate whether the intrinsic functional network connectivity (FNC) with significant between-group differences could differentiate social anxiety disorder (SAD) patients from healthy controls (HC) at the individual level, we performed exploratory support vector machine (SVM) analyses to conduct single-subject classification [1]. A widely used machine learning model, SVM maps the input dataset to a feature space through a set of mathematical functions (kernels), in which the model determines the hyperplane maximizing the margin between different classes in a training set [2,3]. Then SVM can use the classification strategy learned from the training dataset to predict individual classification in testing sample. We used an integrated software, LIBSVM, for support vector classification [4], which proceeds in these steps. First, each subject’s average intra-network FNC of each cluster and inter-network FNC values that showed significant between-group differences were regarded as features for model training. We then utilized a leave-one-out cross-validation scheme to separate training and testing sets: one subject was chosen as a testing set and the remaining sample was the training dataset in each iteration. Before the SVM classification, we carried out data normalization on the feature matrix to guarantee the features were at the same magnitude for subsequent analyses; then we conducted an internal 5-fold stratified nested cross-validation to select the optimal hyperparameter (i.e., the soft margin parameter C, which controls for the balance between increasing separation margin and reducing training errors) in the training set [5]. Specifically, we optimized C by a grid search in the range of 2^-10^ to 2^10^ with step length of 2^0.2^. Once the optimized value of C was determined, it was used in the following analyses. The SVM classification algorithm with a linear kernel was performed to determine the hyperplane in the feature space, and classification strategy that it learned from the training sample was used to predict individual classification in testing dataset. Classification performance was assessed by sensitivity, specificity, and total accuracy based on testing set; a receiver operating characteristic (ROC) curve was constructed, in which the area under the curve (AUC) was calculated for quantification. Finally, nonparametric permutation test was used to estimate statistical significance for the machine learning model [6]; the SVM classification processes were repeated 5000 times with the group labels re-permuted at random; *P* was then calculated as dividing by the number of permutations where classification accuracy based on randomly re-permuted labels was better than that of the real labels.

### Results

**Table S1. Brain regions with significant differences of intrinsic intra-network functional connectivity between SAD patients and HC**

| **Significant clusters** | **RSNs** | **Peak MNI coordinate of significant clusters** | | | **Cluster size (voxels)** | **Peak T value** |
| --- | --- | --- | --- | --- | --- | --- |
|  |  | **X** | **Y** | **Z** |  |  |
| **SAD < HC** | | | | | | |
| L_precuneus | pDMN | -9 | -57 | 57 | 32 | -4.87 |
| L_IPL | AUN | -39 | -30 | 27 | 30 | -4.27 |
| Paracentral/R_precentral gyrus | dSMN | 15 | -30 | 69 | 198 | -4.78 |
| L_ postcentral gyrus | dSMN | -48 | -15 | 54 | 62 | -4.54 |
| L_calcarine | mVN | -12 | -75 | 15 | 80 | -4.04 |
| L_calcarine | mVN | -12 | -45 | 3 | 33 | -4.94 |
| R_calcarine | mVN | 9 | -63 | 18 | 43 | -4.08 |
| R_calcarine | pVN | 12 | -90 | 9 | 128 | -4.51 |
| R_IOG/FFG | lVN | 36 | -72 | -9 | 108 | -4.55 |
| R_ caudate | SCN | 9 | -3 | 18 | 34 | -4.82 |
| **SAD > HC** | | | | | | |
| R_SFG | aDMN | 15 | 45 | 24 | 31 | 4.35 |
| R_STG | AUN | 42 | -9 | -15 | 37 | 4.82 |

All clusters survived correction for multiple comparisons with a significance threshold of a voxel-wise value of *P* < 0.001 and a family-wise error‐corrected *P* < 0.05 at cluster level.

Abbreviations: aDMN, anterior default mode network; AUN, auditory network; dSMN, dorsal sensorimotor network; FFG, fusiform gyrus; HC, healthy controls; IOG, inferior occipital gyrus; IPL, inferior parietal lobe; L, left; lVN, lateral visual network; MNI, Montreal Neurological Institute; mVN, medial visual network; pDMN, posterior default mode network; pVN, posterior visual network; R, right; RSNs, resting-state networks; SAD, social anxiety disorder; SCN, subcortical network; SFG, superior frontal gyrus; STG, superior temporal gyrus.

**Table S2. Single-subject classification of SAD patients versus HC**

| **Features** | **Total Accuracy (%)** | | **Sensitivity (%)** | **Specificity (%)** |
| --- | --- | --- | --- | --- |
| **Intra-network FNC** | 86.7 | 91.3 | | 88.5 |
| **Inter-network FNC** | 69.4 | 63.0 | | 84.6 |
| **Combined intra-/inter-network FNC** | 81.6 | 80.4 | | 84.6 |

Note: total accuracy, sensitivity, and specificity were computed with the SAD group as the positive class. Abbreviations: FNC, functional network connectivity; HC, healthy controls; SAD, social anxiety disorder.

**Figure S1. Clinical relevance of intrinsic FNC.** Scatter plots depicting the correlation between illness duration or symptom severity and intrinsic FNC in the corresponding networks. (A-B) The scores on the x-axis represent the standardized residuals of the duration, while the scores on the y-axis represent the standardized residuals of the intrinsic FNC in the corresponding networks, after sex, age, and mean frame-wise displacement were regressed out. (C-D) The scores on the x-axis represent the standardized residuals of the symptom severity, while the scores on the y-axis represent the standardized residuals of the intrinsic FNC in the corresponding networks, after sex, age, and mean frame-wise displacement were regressed out. Abbreviations: FNC, functional network connectivity; LSAST and LSASA, total score and avoidance factor scores on the Liebowitz Social Anxiety Scale; mVN, medial visual network; SCN, subcortical network; vSMN, ventral sensorimotor network.


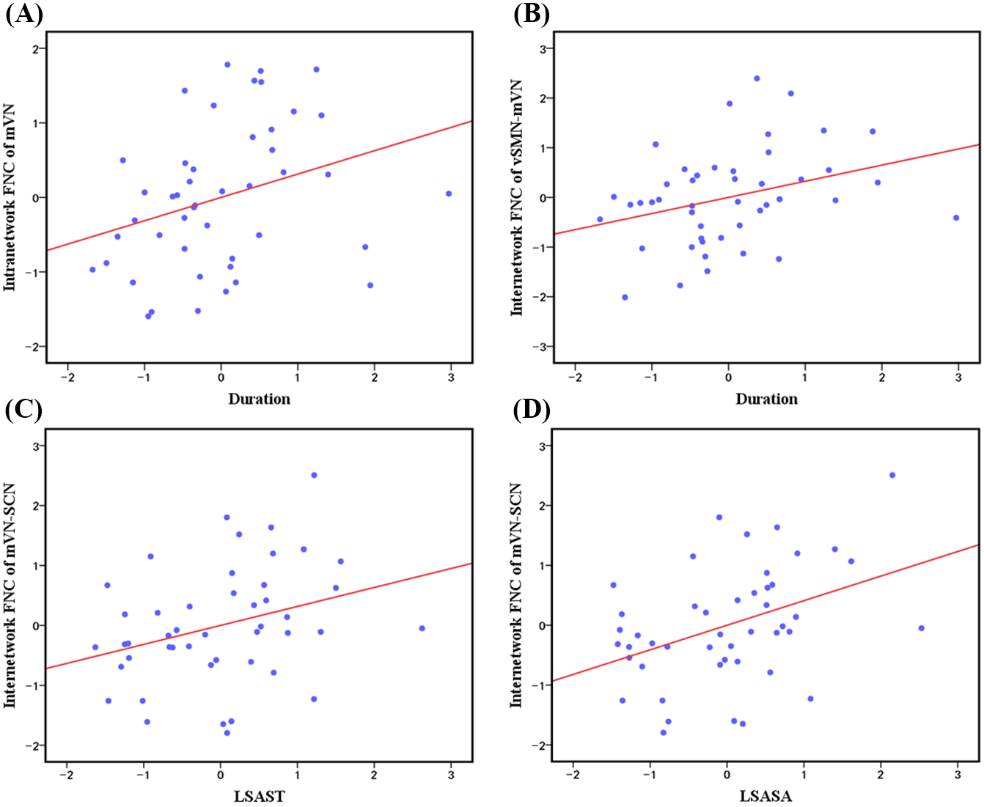


**References**

[1] G Orrù, W Pettersson-Yeo, AF Marquand, G Sartori, A Mechelli. Using support vector machine to identify imaging biomarkers of neurological and psychiatric disease: a critical review. Neurosci Biobehav Rev 2012;36:1140-52.

[2] C Cortes, V Vapnik. Support-vector networks. Machine Learning 1995;20:273-97.

[3] F De Martino, G Valente, N Staeren, J Ashburner, R Goebel, E Formisano. Combining multivariate voxel selection and support vector machines for mapping and classification of fMRI spatial patterns. NeuroImage 2008;43:44-58.

[4] C-C Chang, C-J Lin. LIBSVM: A library for support vector machines. ACM Transactions on Intelligent Systems and Technology 2011;2:1-27.

[5] X Suo, D Lei, W Li, et al. Psychoradiological abnormalities in treatment-naive noncomorbid patients with posttraumatic stress disorder. Depress Anxiety 2022;39:83-91.

[6] P Golland, B Fischl. Permutation tests for classification: Towards statistical significance in image-based studies. Inf Process Med Imaging 2003;18:330-41.
